# Impact of the COVID-19 Pandemic on Liver Cancer Staging at a Multidisciplinary Liver Cancer Clinic

**DOI:** 10.1097/AS9.0000000000000207

**Published:** 2022-10-03

**Authors:** Daniel Li, Angela Y. Jia, Jane Zorzi, Paige Griffith, Amy K. Kim, Doan Dao, Robert A. Anders, Christos Georgiades, Robert P. Liddell, Kelvin Hong, Nilofer S. Azad, Won Jin Ho, Marina Baretti, Eric Christenson, Azarakhsh Baghdadi, Ihab R. Kamel, Jeffrey Meyer, Elie Ghabi, Richard A. Burkhart, Kelly Lafaro, Jin He, Chris Shubert, Mark Yarchoan

**Affiliations:** From the * Department of Oncology, Sidney Kimmel Comprehensive Cancer Center, Johns Hopkins University School of Medicine, Baltimore, MD; † Department of Radiation Oncology and Molecular Radiation Sciences, Johns Hopkins University School of Medicine, Baltimore, MD; ‡ Department of Medicine, Gastroenterology and Hepatology, Johns Hopkins University School of Medicine, Baltimore, MD; § Department of Pathology, Johns Hopkins University School of Medicine, Baltimore, MD; ∥ Department of Radiology and Radiological Sciences, Division of Vascular and Interventional Radiology, Johns Hopkins University School of Medicine, Baltimore, MD; ¶ Department of Radiology and Radiological Sciences, Johns Hopkins University School of Medicine, Baltimore, MD; # Department of Surgery, Johns Hopkins University School of Medicine, Baltimore, MD.

**Keywords:** bile duct cancer, cholangiocarcinoma, COVID-19, hepatocellular carcinoma, liver cancer

## Abstract

To compare liver cancer resectability rates before and during the COVID-19 pandemic.

**Background:**

Liver cancers usually present with nonspecific symptoms or are diagnosed through screening programs for at-risk patients, and early detection can improve patient outcomes. In 2020, the COVID-19 pandemic upended medical care across all specialties, but whether the pandemic was associated with delays in liver cancer diagnosis is not known.

**Methods:**

We performed a retrospective review of all patients evaluated at the Johns Hopkins Multidisciplinary Liver Cancer Clinic from January 2019 to June 2021 with a new diagnosis of suspected or confirmed hepatocellular carcinoma (HCC) or biliary tract cancer (BTC).

**Results:**

There were 456 liver cancer patients (258 HCC and 198 BTC). From January 2019 to March 2020 (pre-pandemic), the surgical resectability rate was 20%. The subsequent 6 months (early pandemic), the resectability rate decreased to 11%. Afterward from October 2020 to June 2021 (late pandemic), the resectability rate increased to 27%. The resectability rate early pandemic was significantly lower than that for pre-pandemic and later pandemic combined (11% lower; 95% confidence interval [CI], 2%–20%). There was no significant difference in resectability rates pre-pandemic and later pandemic (7% difference; 95% CI, –3% to 16%). In subgroup analyses, the early pandemic was associated with a larger impact in BTC resectability rates than HCC resectability rates. Time from BTC symptom onset until Multidisciplinary Liver Clinic evaluation increased by over 6 weeks early pandemic versus pre-pandemic (Hazard Ratio, 0.63; 95% CI, 0.44–0.91).

**Conclusions:**

During the early COVID-19 pandemic, we observed a drop in the percentage of patients presenting with curable liver cancers. This may reflect delays in liver cancer diagnosis and contribute to excess mortality related to the COVID-19 pandemic.

## INTRODUCTION

Liver cancer is the fourth leading cause of cancer-related deaths worldwide and is the most rapidly rising cause of cancer death in the United States.^1–3^ Hepatocellular carcinoma (HCC) is the most common form of primary liver cancer, while biliary tract cancers (BTCs, which include intrahepatic cholangiocarcinoma, extrahepatic cholangiocarcinoma, and gallbladder cancer) represent approximately 15% of primary liver cancers.^1,3^

Early diagnosis of liver cancer is intimately associated with improved patient outcomes. Data from the National Cancer Institute’s Surveillance, Epidemiology, and End Results database indicates that the 5-year relative survival rates for localized liver cancer patients is 34%, while that for liver cancer patients with metastatic spread is 3%.^4^ For cholangiocarcinoma, the 5-year relative survival rate is 25% for localized disease and 2% for metastatic disease.^5^ Cirrhosis is a major risk factor for primary liver cancers, and to facilitate early diagnosis of liver cancer in this at risk population, the American Association for the Study of Liver Diseases and the European Association for the Study of the Liver recommend HCC abdominal ultrasound screening every 6 months for all cirrhotic patients except those of Child-Pugh class C not on a transplant waiting list.^6,7^

The COVID-19 pandemic has disrupted cancer care in the United States. Hospitals have had to reallocate resources to build capacity for increasing numbers of coronavirus admissions,^8,9^ and many nonessential cancer screenings, office visits, and surgeries have had to be canceled.^10,11^ To illustrate, a March 2020 to June 2020 international survey of 91 medical centers found that HCC and BTC screening programs were modified or canceled in over 80% of centers and all but systemic treatments were canceled or delayed in most centers.^12^ Due to COVID-19 delaying liver cancer screening and treatment, some have hypothesized there would eventually be an increase in liver cancer excess deaths due to more patients presenting with higher disease burdens and surgically unresectable disease.^13^

The authors of this report were aware of anecdotes of liver cancer patients who received delayed diagnoses of liver cancer and very advanced disease presentations because of decreased availability of liver screenings, or concerns about interacting with the healthcare system during COVID-19, particularly during the first 6 months of the pandemic. These anecdotes prompted us to perform a retrospective study investigating the presentation of liver cancer at the Johns Hopkins Multidisciplinary Liver and Biliary Cancer Clinic (MDLC) from 2019 to 2021 to determine if the pandemic was associated with more advanced disease presentations of liver cancer. We found that though there was a decrease in liver cancer patients presenting with surgically resectable disease during the beginning of the COVID-19 pandemic, these resectability rates increased to pre-pandemic levels after approximately 6 months.

## METHODS

A retrospective review was conducted using data from the MDLC between January 2019 and June 2021. The inclusion criteria for the study were as follows: (1) new patients who were evaluated at the MDLC, (2) confirmed or strongly suspected HCC or BTC, and (3) age ≥ 18 years. The study protocol was approved by the Johns Hopkins Institutional Review Board (IRB00231803).

### MDLC Workflow

The MDLC includes physicians from hepatology, interventional radiology, medical oncology, palliative care, pathology, radiation oncology, radiology, and hepatobiliary surgical oncology. Previous work from our group described referral patterns and MDLC evaluation algorithms.^14,15^ A full-time triage nurse screened all referrals of suspected or confirmed HCC or BTC, reviewed the patient’s history of disease and pertinent workup, obtained outside imaging and pathological slides for Johns Hopkins Hospital internal review, and assisted with necessary additional workup. All new patients with HCC who were not directly referred for transplant evaluation and patients with BTC that were not extensive stage at Johns Hopkins were initially evaluated at the MDLC. Patients with extensive metastasis were generally seen in medical oncology only and not in MDLC but are still captured by our data. Patients with malignant tumors originating at the ampulla of Vater were usually referred to pancreas multidisciplinary clinic. There were no substantive changes in the staffing of MDLC during the study period; however, the MDLC discussions took place exclusively over video for the study period after March 25, 2020.

### Primary Outcome

The primary outcome surgical resectability was defined as in previous work.^15^ HCC patients were classified into categories broadly based on the 2018 updated Barcelona Clinic Liver Cancer (BCLC) framework.^6^ HCC BCLC A patients (1 lesion or up to 3 lesions each <3 cm and Child-Turcotte-Pugh score A–B) were generally classified as having surgically resectable disease. Nonsurgical HCC BCLC A patients were offered ablation, while larger tumors and/or tumors in difficult locations (hepatic dome, caudate lobe, central biliary tree, abutting adjacent organs, proximal to major blood vessels) were considered for radiation.

BTC patients were classified into 4 groups based on surgical resectability using criteria previously established at our institution^15^: (1) resectable; (2) technically resectable with high-risk features (regional lymph node involvement, biologically at high risk for recurrence); (3) locally advanced (currently unresectable due to liver remnant or vascular involvement concerning for high risk for margin-positive surgical outcome, but potentially explorable after neoadjuvant chemotherapy); and (4) unresectable (invasion into the remnant artery, distant metastases, or insufficient liver remnant precluding future resectability). Assignment to these categories was based on the surgical oncologist’s determination at the time of the multidisciplinary discussion. For the present analysis, patients with disease that was determined to be resectable or technically resectable with high-risk features at the time of MDC presentation were grouped together as resectable; other patients were considered to be unresectable. There were no changes in surgical resectability criteria during the study period.

The United States declared a national emergency for COVID-19 on March 13, 2020, and MDLC multidisciplinary conference and many MDLC patient consultations were transitioned to video/telehealth beginning on March 25, 2020.^16^ After July 29, 2020, most consultations were transitioned back to in person and only a handful of visits were telehealth. Therefore, we defined 3 study period times: pre-pandemic (January 2019 to March 2020), early pandemic at our institution (April 2020 to September 2020, the second and third quarters of 2020), and later pandemic (October 2020 to June 2021).

### Time From Symptoms and Imaging Until MDLC

Time from first documented symptoms (includes abdominal pain, weight loss, pale stools, elevated liver function tests, hyperbilirubinemia, jaundice, pruritus, lymphadenopathy, other skin changes, dark urine, altered mental status, change in bowel habits, weakness, fatigue, nausea, vomiting, or incidental findings on imaging) until MDLC and time from first imaging (Computer Tomography, Magnetic Resonance Imaging, or Endoscopic Retrograde Cholangiopancreatography/Magnetic Resonance Cholangiopancreatography) until MDLC for pre-pandemic and early pandemic BTC patients were extracted using electronic health record chart review.

### Additional Variables and Data

In addition to the outcomes above, age, biologic sex, self-identified race and ethnicity, home state of residence, Eastern Cooperative Oncology Group (ECOG) status, underlying liver disease, prior treatment, and type of provider seen were extracted for each patient. Canceled liver cancer cases and reasons for cancelation from the early and later pandemic study periods (April 2020 to June 2021) were also extracted from the electronic health record system.

### Statistical Methods

To explore how variables may have differed across the 3 time periods, 1-way analysis of variance was used to compare differences in means for continuous variables and Fisher exact test was used to compare differences in distributions of categorical variables. Continuous variables were verified to be approximately normally distributed with equal standard deviations. These obtained *P* values were exploratory in nature and do not indicate statistical significance.

The primary endpoint was defined to be the difference in overall liver cancer surgical resectability rates during the early pandemic versus during the pre-pandemic and later pandemic study periods combined. The point estimate and 95% confidence interval (CI) difference between the combined pre-pandemic and later pandemic rates were calculated. To assess the validity of combining the pre-pandemic and later pandemic periods, a 95% CI of the difference between the pre-pandemic and later pandemic resectability rates were calculated.

We performed additional exploratory analyses looking at surgical resectability rates among HCC and BTC patients separately and at resectability rates by quarter. When comparing pre-pandemic and early pandemic BTC times from symptoms to MDLC and time from imaging to MDLC, Cox’s proportional hazards model was used to assess for differences in hazards. We also performed an exploratory multivariable logistic regression predicting resectability among BTC patients. As a sensitivity analysis, we defined the early pandemic period to be the first 9 months of COVID-19 (rather than first 6 months) from April 2020 to December 2020. The pre-pandemic period was unchanged, and the later pandemic period was from January to June 2021. We performed an additional sensitivity analyses looking at study periods of January 2019 to December 2019, January 2020 to December 2020, January 2021 to June 2021, and April 2021 to June 2021. These study periods approximately correlate with before COVID (2019), the alpha variant and no vaccine (January 2020 to December 2020), 1–2 dose vaccine available (January 2021 to June 2021), and beginning of delta variant (April 2021 to June 2021).

## RESULTS

### Patient Characteristics

A total of 456 liver cancer patients (258 HCC and 198 BTC) were evaluated in the MDLC between January 2019 and June 2021. Table 1 shows HCC patient characteristics for the 3 study periods. Across all 3 study periods, HCC patients were on average 66–68 years old, mostly male, mostly White non-Hispanic, and mostly came from Maryland. A smaller percentage of HCC patients were noncirrhotic or Child-Pugh class A early pandemic (55%) compared with pre-pandemic (74%) and later pandemic (83%). A smaller percentage of HCC patients had an ECOG performance score of 0 early pandemic (30%) compared with pre-pandemic (50%) and later pandemic (47%). Across all 3 study periods, the most common underlying liver disease in HCC patients was hepatitis C (pre-pandemic 51%, early pandemic 51%, later pandemic 47%), most HCC patients had no treatments prior to MDLC (pre-pandemic 69%, early pandemic 66%, later pandemic 72%), and most HCC patients saw a medical oncologist (pre-pandemic 65%, early pandemic 68%, later pandemic 72%). Fewer HCC patients saw a surgeon early pandemic (0%) compared with pre-pandemic (17%) and later pandemic (21%).

**TABLE 1.** HCC Patient Characteristics at Initial Encounter Pre-Pandemic (January 2019 to March 2020), Early Pandemic (April 2020 to September 2020), and Later Pandemic (October 2020 to June 2021)

Variable	HCC (n = 258)			
	Pre-Pandemic (n = 133)	Early Pandemic (n = 47)	Later Pandemic (n = 78)	*P * *
Age, years, mean (SD)	66.3 (9.3)	67.1 (10.0)	67.5 (10.7)	0.695
Sex, male	105 (79%)	34 (72%)	58 (74%)	0.581
Race/ethnicity				
White, non-Hispanic	77 (58%)	23 (49%)	42 (54%)	0.922
Black, non-Hispanic	36 (27%)	18 (38%)	26 (33%)	
Asian, non-Hispanic	6 (5%)	2 (4%)	4 (5%)	
Hispanic, any race	5 (4%)	1 (2%)	1 (1%)	
Other identification	9 (7%)	3 (6%)	5 (6%)	
Home state†				
Maryland	103 (77%)	31 (66%)	55 (71%)	0.462
Bordering MD	25 (19%)	14 (30%)	18 (23%)	
Other	5 (4%)	2 (4%)	5 (6%)	
HCC Barcelona stage				
0 or A	23 (17%)	7 (15%)	25 (32%)	0.143
B	40 (30%)	16 (34%)	24 (31%)	
C	64 (48%)	23 (49%)	28 (38%)	
D	6 (5%)	1 (2%)	1 (1%)	
Child-Pugh				
Not cirrhotic/A	98 (74%)	26 (55%)	65 (83%)	0.013
B	25 (19%)	15 (32%)	11 (14%)	
C	10 (8%)	6 (13%)	2 (3%)	
ECOG				
0	67 (50%)	14 (30%)	37 (47%)	0.005
1	37 (28%)	29 (62%)	33 (42%)	
2	19 (14%)	4 (9%)	6 (8%)	
3	6 (5%)	0 (0%)	2 (3%)	
4	4 (3%)	0 (0%)	0 (0%)	
Underlying liver disease‡				
None	15 (11%)	2 (4%)	6 (8%)	0.360
Hepatitis B	13 (10%)	7 (15%)	7 (9%)	0.540
Hepatitis C	68 (51%)	24 (51%)	37 (47%)	0.865
Nonalcoholic Steatohepatitis/NAFL	18 (14%)	10 (21%)	16 (21%)	0.290
Alcohol	30 (23%)	8 (17%)	21 (27%)	0.476
Other	11 (8%)	3 (6%)	7 (9%)	0.954
Prior treatment				
Loco-regional	26 (20%)	12 (26%)	14 (18%)	0.875
Other	15 (11%)	4 (9%)	8 (10%)	
None	92 (69%)	31 (66%)	56 (72%)	
Provider type seen‡				
Medical oncology	86 (65%)	32 (68%)	56 (72%)	0.570
Surgery	23 (17%)	0 (0%)	16 (21%)	0.001
Interventional radiology	65 (49%)	19 (40%)	43 (55%)	0.286
Radiation oncology	26 (20%)	3 (6%)	10 (13%)	0.084
Palliative care	10 (8%)	5 (11%)	3 (4%)	0.309
Hepatology	75 (56%)	21 (45%)	41 (53%)	0.387

*P values should be interpreted as exploratory and do not indicate statistical significance.

†States bordering MD include Virginia, Washington DC, Delaware, Pennsylvania, and West Virginia.

‡Whereas for the other variables in which patients can only fall into 1 category, a single patient may have multiple liver diseases and seen multiple provider types.

MD indicates Maryland.

We similarly looked at BTC patient characteristics for the 3 study periods. Table 2 shows across all 3 study periods, BTC patients were on average 64 years old, around half male, mostly White non-Hispanic, and most came from Maryland or a state bordering Maryland. BTC cancers were most commonly intrahepatic (pre-pandemic 64%, early pandemic 57%, later pandemic 63%). A smaller percentage of BTC patients had an ECOG performance score of 0 early pandemic (21%) compared with pre-pandemic (40%) and later pandemic (49%). Across all 3 study periods, most BTC patients had no liver disease (pre-pandemic 82%, early pandemic 62%, later pandemic 65%), most BTC patients had no treatments prior to MDLC (pre-pandemic 62%, early pandemic 64%, later pandemic 54%), and most BTC patients at MDLC saw a medical oncologist (pre-pandemic 90%, early pandemic 90%, later pandemic 83%). Fewer BTC patients saw a surgeon early pandemic (10%) compared with pre-pandemic (32%) and later pandemic (23%).

**TABLE 2.** BTC Patient Characteristics at Initial Encounter Pre-Pandemic (January 2019 to March 2020), Early Pandemic (April 2020 to September 2020), and Later Pandemic (October 2020 to June 2021)

Variable	BTC (n = 198)			
	Pre-Pandemic (n = 91)	Early Pandemic (n = 42)	Later Pandemic (n = 65)	*P* *
Age, years, mean (SD)	64.0 (13.1)	63.9 (14.5)	64.0 (12.4)	0.998
Sex, male	52 (57%)	23 (55%)	27 (42%)	0.146
Race/ethnicity				
White, non-Hispanic	65 (71%)	28 (67%)	45 (69%)	0.641
Black, non-Hispanic	11 (12%)	7 (17%)	9 (14%)	
Asian, non-Hispanic	7 (8%)	2 (5%)	5 (8%)	
Hispanic, any race	2 (2%)	3 (7%)	0 (0%)	
Other identification	6 (7%)	2 (5%)	6 (9%)	
Home state†				
Maryland	43 (47%)	30 (71%)	35 (54%)	0.036
Bordering MD	40 (44%)	8 (19%)	20 (31%)	
Other	8 (9%)	4 (10%)	10 (15%)	
BTC resectability group				
Resectable	12 (13%)	1 (2%)	5 (8%)	0.023
Technically resectable with high-risk features	10 (11%)	2 (5%)	10 (15%)	
Locally advanced	35 (38%)	11 (26%)	27 (42%)	
Unresectable	34 (37%)	28 (67%)	23 (35%)	
BTC location				
Intrahepatic	58 (64%)	24 (57%)	41 (63%)	<0.001
Hilar	12 (13%)	10 (24%)	15 (23%)	
Extrahepatic	17 (19%)	0 (0%)	3 (5%)	
Gallbladder	4 (4%)	5 (8%)	5 (8%)	
Unknown	0 (0%)	3 (2%)	1 (2%)	
ECOG				
0	36 (40%)	9 (21%)	32 (49%)	0.124
1	39 (43%)	24 (57%)	27 (42%)	
2	10 (11%)	7 (17%)	5 (8%)	
3	4 (4%)	2 (5%)	1 (2%)	
4	2 (2%)	0 (0%)	0 (0%)	
Underlying liver disease‡				
None	75 (82%)	26 (62%)	42 (65%)	0.943
Hepatitis B	4 (4%)	2 (5%)	2 (3%)	0.794
Hepatitis C	4 (4%)	1 (2%)	4 (6%)	0.655
Nonalcoholic Steatohepatitis/Nonalcoholic Fatty Liver	3 (3%)	2 (5%)	6 (9%)	0.165
Alcohol	4 (4%)	3 (7%)	1 (2%)	0.264
Other	6 (7%)	10 (24%)	10 (15%)	0.003
Prior treatment				
Loco-regional	4 (4%)	1 (2%)	2 (3%)	0.788
Other	31 (34%)	14 (33%)	28 (43%)	
None	56 (62%)	27 (64%)	35 (54%)	
Provider type seen‡				
Medical oncology	82 (90%)	38 (90%)	54 (83%)	0.050
Surgery	29 (32%)	4 (10%)	15 (23%)	0.119
Interventional radiology	21 (23%)	4 (10%)	7 (11%)	0.271
Radiation oncology	22 (24%)	3 (7%)	11 (17%)	0.206
Palliative care	9 (10%)	3 (7%)	5 (8%)	1.000
Hepatology	3 (3%)	1 (2%)	2 (3%)	1.000

*P values should be interpreted as exploratory and do not indicate statistical significance.

†States bordering MD include Virginia, Washington DC, Delaware, Pennsylvania, and West Virginia.

‡Whereas for the other variables in which patients can only fall into 1 category, a single patient may have multiple liver diseases and seen multiple provider types.

MD indicates Maryland.

### Surgical Resectability Rates Declined Early in the Pandemic

**FIGURE 1.**
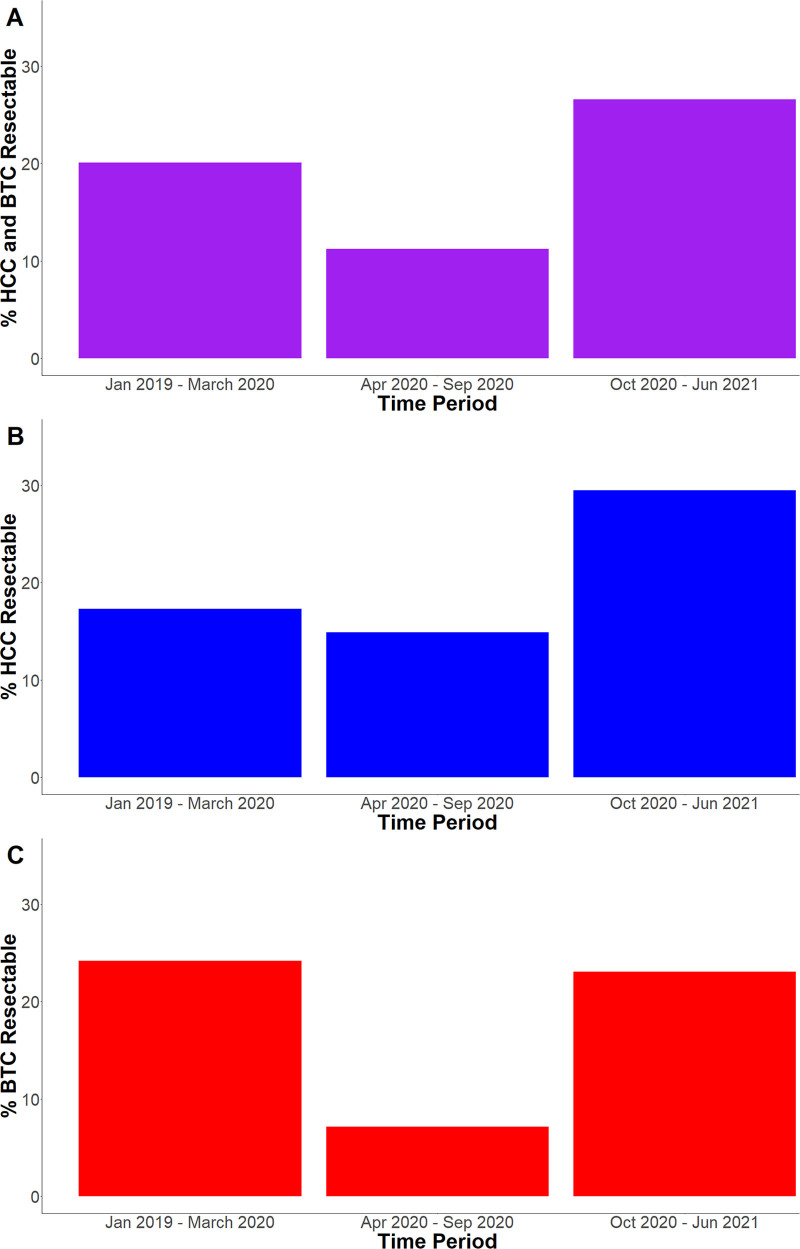
Resectability by Study Period. The percentage of liver cancer patients with resectable disease (A) and by HCC and BTC separately (B and C, respectively) pre-pandemic (January 2019 to March 2020), early pandemic (April 2020 to September 2020), and later pandemic (October 2020 to June 2021).

We looked at resectability rates for all MDLC liver cancer patients by study period. Figure 1A shows pre-pandemic from January 2019 to March 2020, 20% of all liver cancer patients initially presented with surgically resectable disease. During the early pandemic from April 2020 to September 2020 after the United States declared a national COVID emergency, the resectability rate decreased to 11%. Later pandemic from October 2020 to June 2021, the resectability rate increased to 27%. The combined liver cancer resectability rate during the early pandemic was significantly lower than that for the pre-pandemic and later pandemic periods combined (11% lower; 95% CI, 2%–20%). There was no significant difference in combined liver cancer resectability rates between the pre-pandemic and later pandemic periods (7% difference; 95% CI, –3% to 16%).

We further looked at HCC and BTC patients separately. Figure 1B shows pre-pandemic 17% of all HCC patients initially presented with surgically resectable disease. Early pandemic, the resectability rate decreased slightly to 15%. Later pandemic, the resectability rate increased to 29%. The HCC resectability rate during the early pandemic was not significantly lower than that for the pre-pandemic and later pandemic periods combined (7% difference; 95% CI, –6% to 20%). There was no statistically significant difference in HCC resectability rates between the pre-pandemic and later pandemic periods (12% difference; 95% CI, –1% to 25%).

Resectability patterns for BTC patients were different from those of HCC patients. Figure 1C shows pre-pandemic 24% of all BTC patients initially presented with surgically resectable disease. Early pandemic, the resectability rate decreased to 7%. Later pandemic, the resectability rate increased to 23%. The BTC resectability rate during the early pandemic was significantly lower than that for the pre-pandemic and later pandemic periods combined (17% lower; 95% CI, 5%–28%). There was no significant difference in BTC resectability rates between the pre-pandemic and later pandemic periods (1% difference; 95% CI, –14% to 16%).

Across all 3 periods, intrahepatic BTC was most common (pre-pandemic 64%, early pandemic 57%, later pandemic 63%). There were fewer hilar BTC pre-pandemic (13%) compared with early pandemic (24%) and later pandemic (23%). There were more extrahepatic BTC pre-pandemic (19%) compared with early pandemic (0%) and later pandemic (5%). Among all 40 resectable BTC patients, 21 had intrahepatic BTC, 9 had hilar BTC, 5 had extrahepatic BTC, and 5 had gallbladder BTC.

To investigate whether the observed rise in liver cancer resectability rates later pandemic may have been from delayed cases during the early pandemic study period, we extracted canceled liver cancer cases during the early and later pandemic study periods (April 2020 to June 2021). During the early pandemic period, there was only 1 canceled liver cancer case (reason no explanation given). During the later pandemic period, there were only 3 canceled liver cancer cases (no reason specified, patient sick, and needs further evaluation). Given only 1 case was canceled/delayed during the early pandemic period, there was likely an actual rise in resectability rate during the later pandemic.

### Surgical Resectability Rates—Quarterly Trends

**FIGURE 2.**
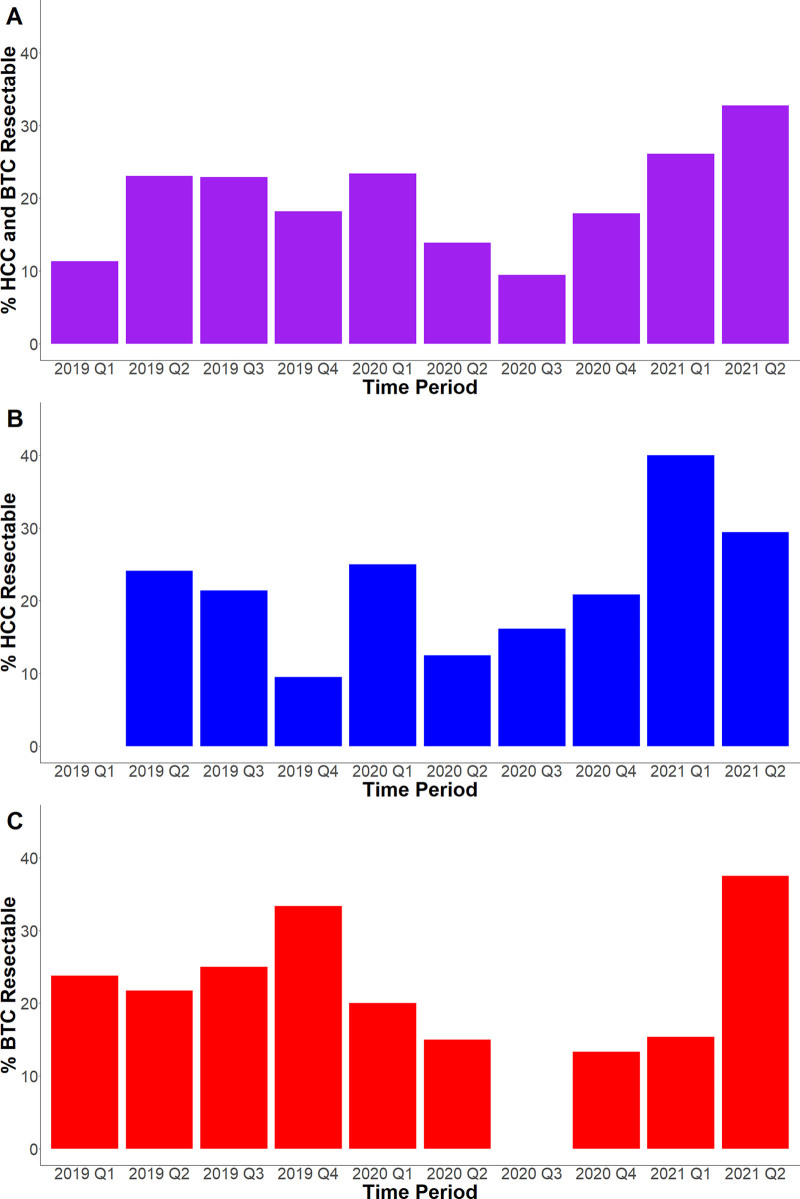
Resectability by Quarter. The percentage of HCC and BTC patients with resectable disease combined (A) and separately (B and C, respectively) by quarter between January 2019 and June 2021.

We further looked at surgical resectability rates by quarter to look at trends at a more granular level. Figure 2A shows quarterly surgical resectability rates for all liver cancer patients. There were smaller combined liver cancer resectability rates in 2019 Q1 (11%), although resectability rates were then higher and remained stable from 2019 Q2 to 2020 Q1 (23%, 23%, 18%, and 23%). With the start of COVID, there was then a gradual decrease in resectability rates 2020 Q2 to Q3 (14% and 9%). After the first 6 months of COVID, there were increasing resectability rates from 2020 Q4 to 2021 Q2 (18%, 26%, and 33%).

We also looked at quarterly surgical resectability trends for HCC and BTC patients separately. Figure 2B shows there was significant variability in HCC resectability rates prior to COVID from 2019 Q1 to 2020 Q1 (0%, 24%, 21%, 9%, and 25%). With the start of COVID from 2020 Q2 to Q4, HCC resectability rates were within the same range of values compared with before COVID (13%, 16%, and 21%). HCC resectability rates were higher from 2021 Q1 to Q2 (40% and 29%).

The quarterly surgical resectability trends for BTC patients were different from those of HCC patients. Figure 2C shows quarterly BTC resectability rates from 2019 Q1 to 2020 Q1 were consistently higher than 20% prior to COVID (24%, 22%, 25%, 33%, and 20%). With the arrival of COVID, BTC resectability rates were then lower from 2020 Q2 to 2021 Q1 (15%, 0%, 13%, 15%). BTC resectability rates were higher for 2021 Q2 (38%).

### Time From Symptom Onset Until MDLC Evaluation Increased Early Pandemic for BTC Patients

The decrease in liver cancer resectability at the beginning of COVID appeared to be driven primarily by a decline in BTC resectability rates, and most patients with BTC are not enrolled in screening programs. Hence, we hypothesized that delays in the medical workup of symptomatic BTC during early COVID-19 may be responsible for the observed decline in BTC resectability. We therefore compared pre-pandemic versus early pandemic versus later pandemic BTC time from symptom onset until MDLC evaluation and time from first imaging until MDLC evaluation using a retrospective review of patient-reported dates of symptoms onset in the MDLC database.

The median time from BTC symptom onset until MDLC evaluation for all BTC patients was pre-pandemic 88 days (interquartile range [IQR], 35–88 days), early pandemic 132 days (IQR, 45–285 days), and later pandemic 65 days (IQR, 40–129 days). There was a statistically significant difference in hazards for time from symptoms until MDLC evaluation early pandemic versus pre-pandemic (Hazard Ratio (HR), 0.63; 95% CI, 0.44–0.91), but not between later pandemic versus pre-pandemic (HR, 1.02; 95% CI, 0.74–1.30).

The median time from first imaging until MDLC evaluation was pre-pandemic 46 days (IQR, 26–113 days), early pandemic 40 days (IQR, 20–195 days), and later pandemic 46 days (IQR, 27–107 days). There were no statistically significant differences in the hazards for time from first imaging until MDLC evaluation early pandemic versus pre-pandemic (HR, 0.87; 95% CI, 0.60–1.25) and later pandemic versus pre-pandemic (HR, 1.01; 95% CI, 0.74–1.39).

### Time From Symptom Onset Until MDLC Evaluation Partly Controls for Changes in BTC Resectability Rates by Study Period

We performed multivariable logistic regression to further explore the association between the variables above and BTC surgical resectability. Table 3 shows unadjusted, 2 partially adjusted, and fully adjusted model results looking at the association between the pandemic study period and BTC surgical resectability. In the unadjusted model, a patient in the early pandemic had decreased odds of having resectable disease compared with a patient pre-pandemic (odds ratio [OR], 0.27; 95% CI, 0.06–0.86). Even after adjusting for age, sex, self-identified race/ethnicity, home state, BTC location, ECOG status, liver disease, and prior treatment, the early pandemic was still associated with decreased odds of having resectable disease (OR, 0.26; 95% CI, 0.06–0.86).

**TABLE 3.** Multivariable Logistic Regression of BTC Resectability

Variable	Odds Ratio (95% CI)*			
	Unadjusted	Model 1	Model 2	Fully Adjusted
Period				
Pre-pandemic	Reference	Reference	Reference	Reference
Early pandemic	**0.24 (0.05–0.75**)	**0.22 (0.05–0.74**)	0.31 (0.07–1.10)	1.04 (0.18–5.09)
Later pandemic	0.94 (0.44–1.98)	1.17 (0.51–2.63)	1.20 (0.50–2.86)	2.50 (0.79–8.58)
Age (every 10 years)	—	0.89 (0.67–1.20)	0.87 (0.64–1.20)	0.84 (0.56–1.26)
Sex, male	—	1.28 (0.59–2.81)	1.26 (0.56–2.86)	1.14 (0.50–3.26)
Race/ethnicity				
White, non-Hispanic	Reference	Reference	Reference	Reference
Black, non-Hispanic	—	0.55 (0.14–1.74)	0.52 (0.13–1.70)	0.34 (0.06–1.54)
Other	—	0.73 (0.22–2.05)	0.83 (0.24–2.48)	1.83 (0.42–7.16)
Home state, outside MD	—	0.83 (0.38–1.79)	0.87 (0.38–1.98)	0.66 (0.22–1.84)
BTC location, not intrahepatic	—	2.01 (0.92–4.42)	2.22 (0.97–5.15)	2.02 (0.69–6.06)
ECOG, 2–3	—	1.37 (0.50–3.54)	1.24 (0.42–3.42)	2.06 (0.53–8.10)
Liver disease present	—	0.64 (0.25–1.55)	0.55 (0.20–1.40)	0.45 (0.13–1.47)
Prior treatment received	—	**0.43 (0.18–0.98**)	0.67 (0.25–1.68)	0.70 (0.19–2.33)
Time from symptom onset until MDLC (every 30 days)	—	—	**0.76 (0.60–0.90**)	**0.70 (0.52–0.87**)
Seen by surgery	—	—	—	**34.7 (11.8–123**)

*OR > 1 indicates an increased odds of having resectable disease, and bold indicates the 95% confidence interval does not include 1.00.

— indicates that the variable was not included in the model

However, after additionally controlling for time from symptom onset until MDLC, there was no statistically significant difference in odds between the early and pre-pandemic (OR, 0.31; 95% CI, 0.07–1.10). In the fully adjusted model after additionally adding whether a patient saw a surgeon on the initial MDLC visit, there was still no significant association between the early and pre-pandemic (OR, 1.04; 95% CI, 0.18–5.09). The only significant associations were that every 30-day increase in time from symptoms until MDLC was associated with a decreased odds of having resectable disease (OR, 0.70; 95% CI, 0.52–0.87) and that having seen a surgeon was associated with an increased odds of having resectable disease (OR, 34.7; 95% CI, 11.8–123). The large OR for having seen a surgeon is reasonable as 58% of BTC patients who saw a surgeon on the first MDLC visit initially had resectable disease, while only 7% of BTC patients who did not see a surgeon on the first MDLC visit had resectable disease, which corresponds with a raw OR of approximately 20.

To emphasize, these subgroup results presented in Table 3 were performed only on BTC patients and are not applicable to HCC patients.

### Sensitivity Analyses

We performed a sensitivity analysis defining early pandemic as the first 9 months beginning April 2020 rather than the first 6 months. Figure S1A (http://links.lww.com/AOSO/A167) shows resectability rates for all MDLC liver cancer patients, Figure S1B (http://links.lww.com/AOSO/A167) for HCC patients, and Figure S1C (http://links.lww.com/AOSO/A167) shows rates for BTC patients during these modified study periods. Table S1 (http://links.lww.com/AOSO/A167) summarizes results comparing the 6 and 9 months early pandemic study period analyses and provides counts of resectable and total cases for each study period. Results were similar using both study period lengths. Table S2 (http://links.lww.com/AOSO/A167) provides counts of resectable and total cases for each quarter during the study period. The average number of total liver cancer cases per quarter were 45 pre-pandemic, 45 early pandemic, and 48 later pandemic. The average number of resectable cases per quarter were 9 pre-pandemic, 5 early pandemic, and 13 later pandemic. Table S3 (http://links.lww.com/AOSO/A167) provides counts for the modified study periods corresponding to different variants and vaccine availability. Using these modified study periods, surgical resectability rates were lower during the beginning of the pandemic when there was no vaccine and the alpha variant was prevalent compared with later periods when the vaccine was available and the delta variant was more prevalent.

## DISCUSSION

We performed a retrospective analysis to study the impact of the COVID-19 pandemic on liver cancer patients presenting at the Johns Hopkins Hospital MDLC. We found that surgical resectability rates for liver cancer patients were lower during the initial phase of the COVID-19 pandemic but recovered to pre-COVID levels after approximately 6 months. This decrease in liver cancer resectability at the beginning of COVID was primarily driven by BTC resectability rates, and we observed a longer duration between the onset of symptoms in patients with BTC and presentation to the MDLC during early COVID-19.

Our findings add to a growing body of evidence that COVID-19 has disrupted cancer screenings, encounters, and new diagnoses for all types of cancers. A study of US Medicare cancer patients found that in April 2020, screenings for breast, colon, prostate, and lung cancers were lower by 85%, 75%, 74%, and 56%, respectively, compared with the same period in 2019.^17^ An analysis of the COVID and Cancer Research Network, which includes electronic medical record data on over 28 million US patients, also found similar decreases in screening rates of breast cancer and colorectal cancer, along with approximately 39% fewer lung, colorectal, and hematologic cancer encounters and 48%–52% fewer breast, prostate, and melanoma cancer encounters in January 2020 to April 2020 compared with the same months in 2019.^18^ Cross-sectional studies of US patients who received testing at Quest Diagnostics found significantly fewer newly diagnosed patients with breast, colorectal, lung, pancreatic, cervical, gastric, esophageal, and prostate cancer in March 2020 to May 2020 and November 2020 to March 2021 compared with January 2019 to February 2020.^19,20^ Many experts have hypothesized that these disruptions in care and delays in diagnoses might lead to increases in advanced cancer presentations and further excess deaths.^21–23^ Our results are consistent with the first part of this prediction in that we did observe a decrease in surgical resectability among liver cancer patients at the beginning of the COVID-19 pandemic, particularly among BTC patients.

There were no surgical structural or staffing changes during the early pandemic period, and fewer surgeons saw patients in MDLC during this time because of this decrease in resectable disease. Overall, liver cancer cases did not see a significant reduction in capacity during the pandemic. From March 1, 2020, to March 1, 2022, only 14 liver cancer cases were postponed or canceled. Of these postponed or canceled cases, only 1 occurred from March 1, 2020, to September 31, 2020 (early pandemic), 3 occurred from October 1, 2020, to June 30, 2021 (later pandemic), and 10 occurred from July 1, 2021, to March 1, 2022 (after study period).

The return of surgical resectability rates to pre-COVID levels after approximately 6 months was an unexpected finding. In subgroup analyses, this pattern was more pronounced in BTC patients as HCC staging was more consistent during the pandemic. This may reflect that BTC often presents with nonspecific complaints, whereas more HCC patients are identified with routine surveillance that may have been less disrupted throughout the pandemic.^6,7,24^ To elaborate, during the beginning of the pandemic as institutions were transitioning to telehealth, there were likely some growing pains for providers and patients alike. This transition period may have then led to more delays in working up nonspecific symptoms for BTC patients. This hypothesis is supported by our results showing an increase in time from symptom onset until MDLC for BTC patients during the early pandemic versus pre-pandemic and later pandemic. However, because most cirrhotic patients are recommended to have screening abdominal ultrasounds every 6 months, detection of HCC may have been less disrupted during these transitions to telehealth during the early pandemic. As the pandemic continued, patients and their providers may have both become more comfortable with telehealth interactions and new healthcare protocols, leading to expedited workups for nonspecific complaints and quicker detection of BTC cancer.^25^ Although the COVID-19 pandemic is ongoing at the time of this report, one can interpret the recovery of liver cancer resectability rates (particularly among BTC patients) during the study period as a reflection of the resiliency and adaptability of healthcare systems operations. After the early pandemic, there may have also been some catch-up on the backlog of screening and workup of patients with symptomatic disease as well.

Future work should longitudinally follow liver cancer patients presenting during the COVID-19 pandemic to determine whether long-term survival times and outcomes are affected. Future work may also develop screening guidelines for BTC similar to those for HCC to prevent potential decreases in resectability rates in the event of future healthcare disruptions. Strengths of this study include the use of a rigorously annotated cohort of liver cancer patients. Limitations of this analysis include its retrospective nature and that only patients at a single academic medical center were included. Larger database studies are also needed to investigate trends on larger regional and geographic areas. More critically, it is not possible to exclude that changes in resectability rates reflect changes in referral patterns, staging practices in our MDLC, screening practices, or other factors that are unrelated to COVID-19, rather than differences in the diagnosis of liver cancer. In summary, we found that fewer liver cancer patients presented with surgically resectable disease during the beginning of COVID-19 but recovered to pre-pandemic levels by 2021. As an early diagnosis of liver cancer allows for application of potentially curative therapy, and we propose that these trends in the early part of the pandemic may eventually lead to increased liver cancer mortality from COVID-19.

## Acknowledgments

D.L. and A.Y.J. made substantial contributions to analysis of the data. A.Y.J. and J.Z. made substantial contributions to acquisition of the data. D.L. made substantial contributions to drafting the article. All authors made substantial contributions to conception and design and analysis and interpretation of the data, revised the article critically, and gave final approval to the version to be published.

## Supplementary Material



## References

[1] Akinyemiju T Abera S Ahmed M; Global Burden of Disease Liver Cancer Collaboration. The burden of primary liver cancer and underlying etiologies from 1990 to 2015 at the global, regional, and national level: results from the Global Burden Of Disease Study 2015. JAMA Oncol. 2017;3:1683. 28983565 10.1001/jamaoncol.2017.3055 PMC5824275

[2] Bray F Ferlay J Soerjomataram I. Global cancer statistics 2018: GLOBOCAN estimates of incidence and mortality worldwide for 36 cancers in 185 countries. CA Cancer J Clin. 2018;68:394–424. 30207593 10.3322/caac.21492

[3] Petrick JL McGlynn KA. The changing epidemiology of primary liver cancer. Curr Epidemiol Rep. 2019;6:104–111. 31259140 10.1007/s40471-019-00188-3 PMC6599615

[4] American Cancer Society. Liver Cancer Survival Rates. 2021. Available at: https://www.cancer.org/cancer/liver-cancer/detection-diagnosis-staging/survival-rates.html. Accessed December 30, 2021.

[5] American Cancer Society. Survival Rates for Bile Duct Cancer. 2021. Available at: https://www.cancer.org/cancer/bile-duct-cancer/detection-diagnosis-staging/survival-by-stage.html. Accessed December 30, 2021.

[6] Galle PR Forner A Llovet JM, et al. EASL clinical practice guidelines: management of hepatocellular carcinoma. J Hepatol. 2018;69:182–236. 29628281 10.1016/j.jhep.2018.03.019

[7] Marrero JA Kulik LM Sirlin CB. Diagnosis, staging, and management of hepatocellular carcinoma: 2018 practice guidance by the American Association for the Study of Liver Diseases. Hepatology. 2018;68:723–750. 29624699 10.1002/hep.29913

[8] Emanuel EJ Persad G Upshur R. Fair allocation of scarce medical resources in the time of COVID-19. N Engl J Med. 2020;382:2049–2055. 32202722 10.1056/NEJMsb2005114

[9] Hempel S Burke RV Hochman M. Resource Allocation and Pandemic Response: An Evidence Synthesis To Inform Decision Making. Agency for Healthcare Researh and Quality (AHRQ); 2020. 33054151

[10] Riera R Bagattini ÂM Pacheco RL. Delays and disruptions in cancer health care due to COVID-19 pandemic: systematic review. JCO Glob Oncol. 2021;7:311–323. 33617304 10.1200/GO.20.00639 PMC8081532

[11] Al-Quteimat OM Amer AM. The impact of the COVID-19 pandemic on cancer patients. Am J Clin Oncol. 2020;43:452–455. 32304435 10.1097/COC.0000000000000712 PMC7188063

[12] Muñoz-Martínez S Sapena V Forner A. Assessing the impact of COVID-19 on liver cancer management (CERO-19). JHEP Rep. 2021;3:100260. 33644725 10.1016/j.jhepr.2021.100260 PMC7901294

[13] Chan SL Kudo M. Impacts of COVID-19 on liver cancers: during and after the pandemic. Liver Cancer. 2020;9:491–502. 33078127 10.1159/000510765 PMC7490489

[14] Zhang J Mavros MN Cosgrove D. Impact of a single-day multidisciplinary clinic on the management of patients with liver tumours. Curr Oncol. 2013;20:e123–e131. 23559879 10.3747/co.20.1297 PMC3615863

[15] Jia AY Popovic A Mohan AA. Development, practice patterns, and early clinical outcomes of a multidisciplinary liver cancer clinic. Cancer Control. 2021;28:10732748211009945. 33882707 10.1177/10732748211009945 PMC8204642

[16] The White House. A Letter on the Continuation of the National Emergency Concerning the Coronavirus Disease 2019 (COVID- 19) Pandemic. 2021. Available at: https://www.whitehouse.gov/briefing-room/presidential-actions/2021/02/24/notice-on-the-continuation-of-the-national-emergency-concerning-the-coronavirus-disease-2019-covid-19-pandemic/. Accessed December 31, 2021.

[17] Patt D Gordan L Diaz M. Impact of COVID-19 on cancer care: how the pandemic is delaying cancer diagnosis and treatment for american seniors. JCO Clin Cancer Inform. 2020;4:1059–1071. 33253013 10.1200/CCI.20.00134 PMC7713534

[18] London JW Fazio-Eynullayeva E Palchuk MB. Effects of the COVID-19 pandemic on cancer-related patient encounters. JCO Clin Cancer Inform. 2020;4:657–665. 32716647 10.1200/CCI.20.00068 PMC7444638

[19] Kaufman HW Chen Z Niles J. Changes in the number of US patients with newly identified cancer before and during the coronavirus disease 2019 (COVID-19) pandemic. JAMA Netw Open. 2020;3:e2017267. 32749465 10.1001/jamanetworkopen.2020.17267 PMC7403918

[20] Kaufman HW Chen Z Niles JK. Changes in newly identified cancer among US patients from before COVID-19 through the first full year of the pandemic. JAMA Netw Open. 2021;4:e2125681. 34463748 10.1001/jamanetworkopen.2021.25681 PMC8408670

[21] Maringe C Spicer J Morris M. The impact of the COVID-19 pandemic on cancer deaths due to delays in diagnosis in England, UK: a national, population-based, modelling study. Lancet Oncol. 2020;21:1023–1034. 32702310 10.1016/S1470-2045(20)30388-0 PMC7417808

[22] The Lancet Oncology. COVID-19 and cancer: 1 year on. Lancet Oncol. 2021;22:411. 33794199 10.1016/S1470-2045(21)00148-0 PMC8007133

[23] American Society for Radiation Oncology. Results of a National Physician Survey by the American Society for Radiation Oncology. https://www.astro.org/ASTRO/media/ASTRO/News%20and%20Publications/PDFs/ASTRO_COVID19Survey_2021.pdf. Accessed November 24, 2021.

[24] Mehta N Parikh ND Kelley RK. Surveillance and monitoring of hepatocellular carcinoma during the COVID-19 pandemic. Clin Gastroenterol Hepatol. 2021;19:1520–1530. 32652308 10.1016/j.cgh.2020.06.072 PMC7342037

[25] Burbury K Wong ZW Yip D. Telehealth in cancer care: during and beyond the COVID-19 pandemic. Intern Med J. 2021;51:125–133. 33572014 10.1111/imj.15039 PMC8014764

