# Supplementary material for: Impact of the COVID-19 Pandemic on Liver Cancer Staging at a Multidisciplinary Liver Cancer Clinic
Source: Ann Surg Open. 2022 Oct 3;3(4):e207. doi: 10.1097/AS9.0000000000000207 (PMC9782462; doi:10.1097/AS9.0000000000000207)

Table S1. Comparing resectability rates using a 6 versus 9 month early pandemic study period length.

	Early Pandemic Period Length	
	6 Months	9 Months
Combined HCC and BTC		
Resectability Rate		
Pre-Pandemic	20% (45/224)	20% (45/224)
Early Pandemic	11% (10/89)	13% (17/128)
Later Pandemic	27% (38/143)	30% (31/104)
Difference, 95% CI		
Early vs Pre and Later combined	**-11% (-20%, -2%)**	**-10% (-18%, -2%)**
Pre vs Later	-7% (-16%, 3%)	-10% (-21%, 1%)
HCC Only		
Resectability Rate		
Pre-Pandemic	17% (23/133)	17% (23/133)
Early Pandemic	15% (7/47)	17% (12/71)
Later Pandemic	29% (23/78)	33% (18/54)
Difference, 95% CI		
Early vs Pre and Later combined	-7% (-20%, 6%)	-5% (-17%, 6%)
Pre vs Later	-12% (-25%, 1%)	**-16% (-31%, -1%)**
BTC Only		
Resectability Rate		
Pre-Pandemic	24% (22/91)	24% (22/91)
Early Pandemic	7% (3/42)	9% (5/57)
Later Pandemic	23% (15/65)	26% (13/50)
Difference, 95% CI		
Early vs Pre and Later combined	**-17% (-28%, -5%)**	**-16% (-28%, -5%)**
Pre vs Later	1% (-14%, 16%)	-2% (-18%, 15%)
**Bold** indicates 95% CI does not include 0		

Table S2. Counts of liver cancer patients and resectable disease by quarter.

	All Liver Cancer		HCC		BTC	
	Resectable Cases	Total Cases	Resectable Cases	Total Cases	Resectable Cases	Total Cases
2019 Q1	5	44	0	23	5	21
2019 Q2	12	52	7	29	5	23
2019 Q3	11	48	6	28	5	20
2019 Q4	6	33	2	21	4	12
2020 Q1	11	47	8	32	3	15
2020 Q2	5	36	2	16	3	20
2020 Q3	5	53	5	31	0	22
2020 Q4	7	39	5	24	2	15
2021 Q1	12	46	8	20	4	26
2021 Q2	19	58	10	34	9	24

Table S3. Counts of liver cancer patients and resectable disease by modified study periods.

	All Liver Cancer	HCC	BTC
Jan 2019 – Dec 2019 (pre-COVID)	34/177 (19%)	15/101 (15%)	19/76 (25%)
Jan 2020 – Dec 2020 (alpha variant, no vaccine)	28/175 (16%)	20/103 (19%)	8/72 (11%)
Jan 2021 – Jun 2021 (vaccine)	31/104 (30%)	18/54 (33%)	13/50 (26%)
Apr 2021 – Jun 2021 (delta variant)	19/58 (33%)	10/34 (29%)	9/24 (38%)

Figure S1. Sensitivity analysis of 9 month early pandemic period length of the percentage of HCC and BTC patients with resectable disease combined (**A**) and separately (**B** and **C**) pre-pandemic (Jan 2019-Mar 2020), early pandemic (Apr 2020-Dec 2020), and later pandemic (Jan 2021-Jun 2021).

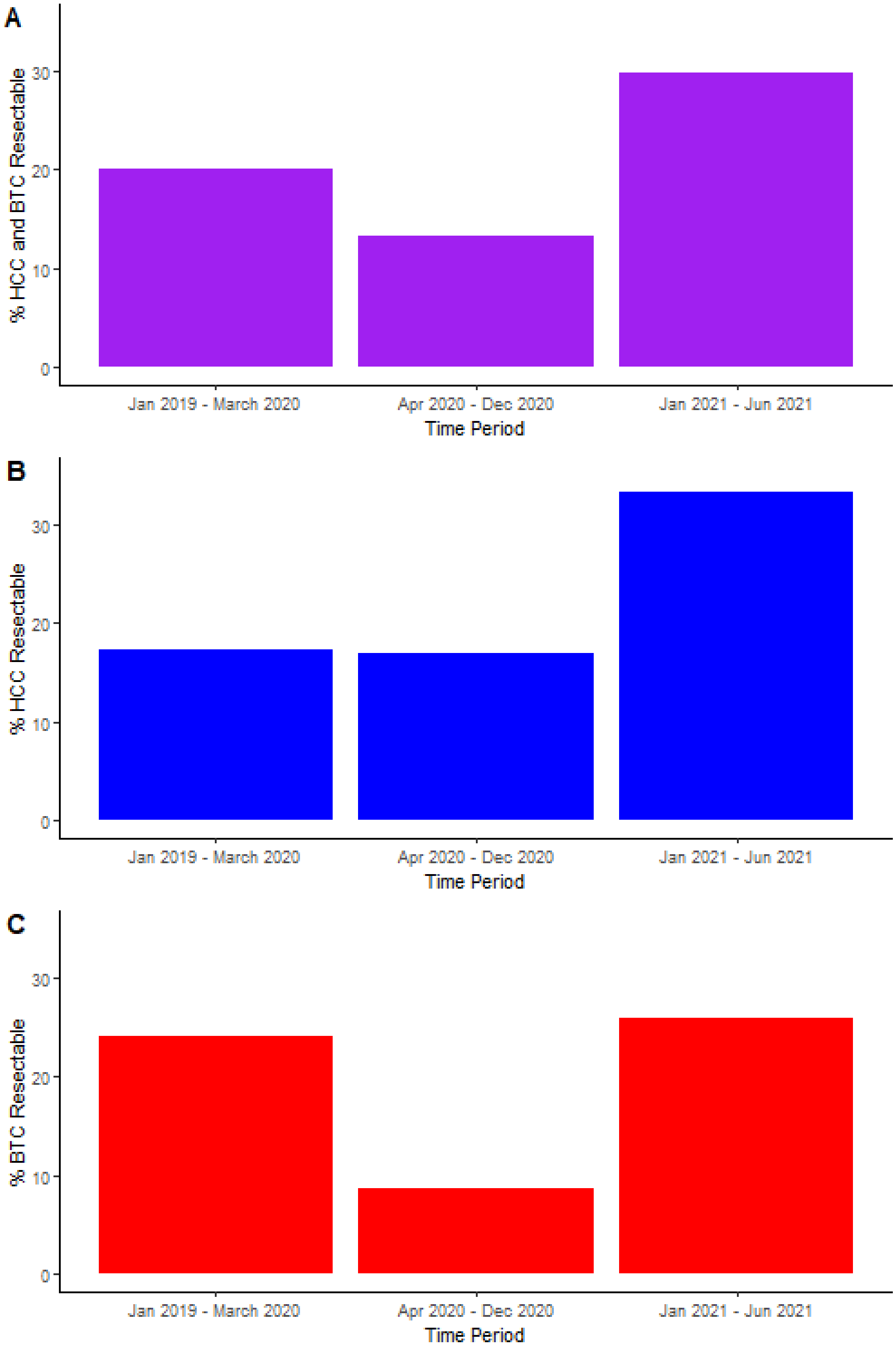

Supplement: Supplementary file 1 [file as9-3-e207-s001.pdf]
